# Cutaneous Metastasis in Patient With Relapse Case of Primary Testicular Lymphoma

**DOI:** 10.1002/ccr3.70720

**Published:** 2025-07-27

**Authors:** Mahesh Mathur, Sumit Paudel, Sambidha Karki, Sandhya Regmi, Shilpa Maharjan, Nabita Bhattarai

**Affiliations:** ^1^ Department of Dermatology College of Medical Sciences Bharatpur Nepal

**Keywords:** cutaneous metastasis, immunohistochemical markers, primary testicular lymphoma, relapse

## Abstract

Non‐Hodgkin's lymphoma originates in the lymphatic system, specifically affecting B or T lymphocytes. Non‐Hodgkin's lymphoma of the testis is highly aggressive extranodal lymphoma originating from the testicles, constituting 1%–2% of all non‐Hodgkin's lymphoma cases. Relapse is reported in non‐Hodgkin's lymphoma of the testes, commonly in the contralateral testis and central nervous system, occurring within 1–2 years from diagnosis of primary lymphoma. Skin metastases occur in 6% to 13% of testicular lymphoma. Diffuse large B‐cell lymphoma is the most common type of testicular lymphoma, typically expressing CD19, CD20, CD22, CD79a, and PAX5 markers. Hereby, we have presented a case of cutaneous metastasis in a patient who underwent remission for 8 years following the treatment of primary testicular lymphoma. Identical immunohistochemical markers from the testis and skin lesion suggested cutaneous metastasis of testicular B cell lymphoma.

## Graphical abstract

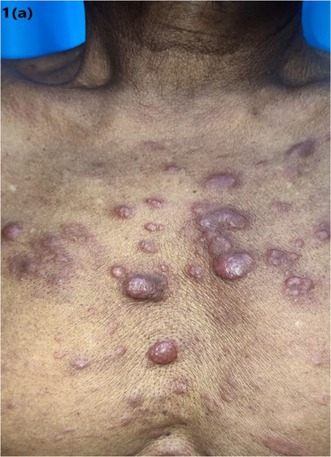

A 72 years male with cutaneous metastatic lesions presenting as multiple erythematous papules and plaques over chest. He had past history of primary testicular lymphoma and was treated with orchidectomy and chemotherapy.

**Abbreviations**
18F‐FDG PET‐CT: 18F‐fluorodeoxyglucose positron emission tomography‐computed tomography
CECT: contrast enhanced computed tomography
CNS: central nervous system
DLBCL: diffuse large B cell lymphoma
HPE: histopathological examination
IHC: immunohistochemistry
LDH: lactate dehydrogenase
NHL: non‐Hodgkin's lymphoma
PCDLBCL: primary cutaneous diffuse large B‐cell lymphoma
PTL: primary testicular lymphoma

**Summary**

- Primary testicular lymphoma is a rare and highly aggressive extranodal lymphoma arising from the testis.
- It has a high relapse rate in the contralateral testis and central nervous system.
- Reports of skin metastasis are rare.
- CD20, CD10, BCL6, BCL2, c‐MYC, MUM‐1, and PAX5 are immunohistochemistry markers of diffuse large B cell lymphoma.

## 1 Introduction

Primary testicular lymphoma (PTL) is a rare extranodal lymphoma originating from the testicles, accounting for 1%–2% of all non‐Hodgkin's lymphoma (NHL), with 80%–98% of these classified as diffuse large B‐cell lymphoma (DLBCL). It is a highly aggressive tumor with a poor prognosis and typically affects the elderly patient [1]. Testicular B‐cell lymphoma (TBCL) has a high relapse rate, with recurrence commonly observed in the contralateral testis and central nervous system (CNS). Other sites of relapse are bone, bone marrow, lungs, and adrenal glands [2]. Skin metastasis is rare and occurs in 6% to 13% of testicular lymphoma cases [3].

Histopathological examination (HPE) of skin biopsy with identical morphological and immunohistochemical (IHC) markers for primary lymphoma confirms the diagnosis of cutaneous metastasis [3]. This case is reported because of the rarity of the malignancy and its metastasis in skin.

## 2 Case History and Examination

**FIGURE 1:**
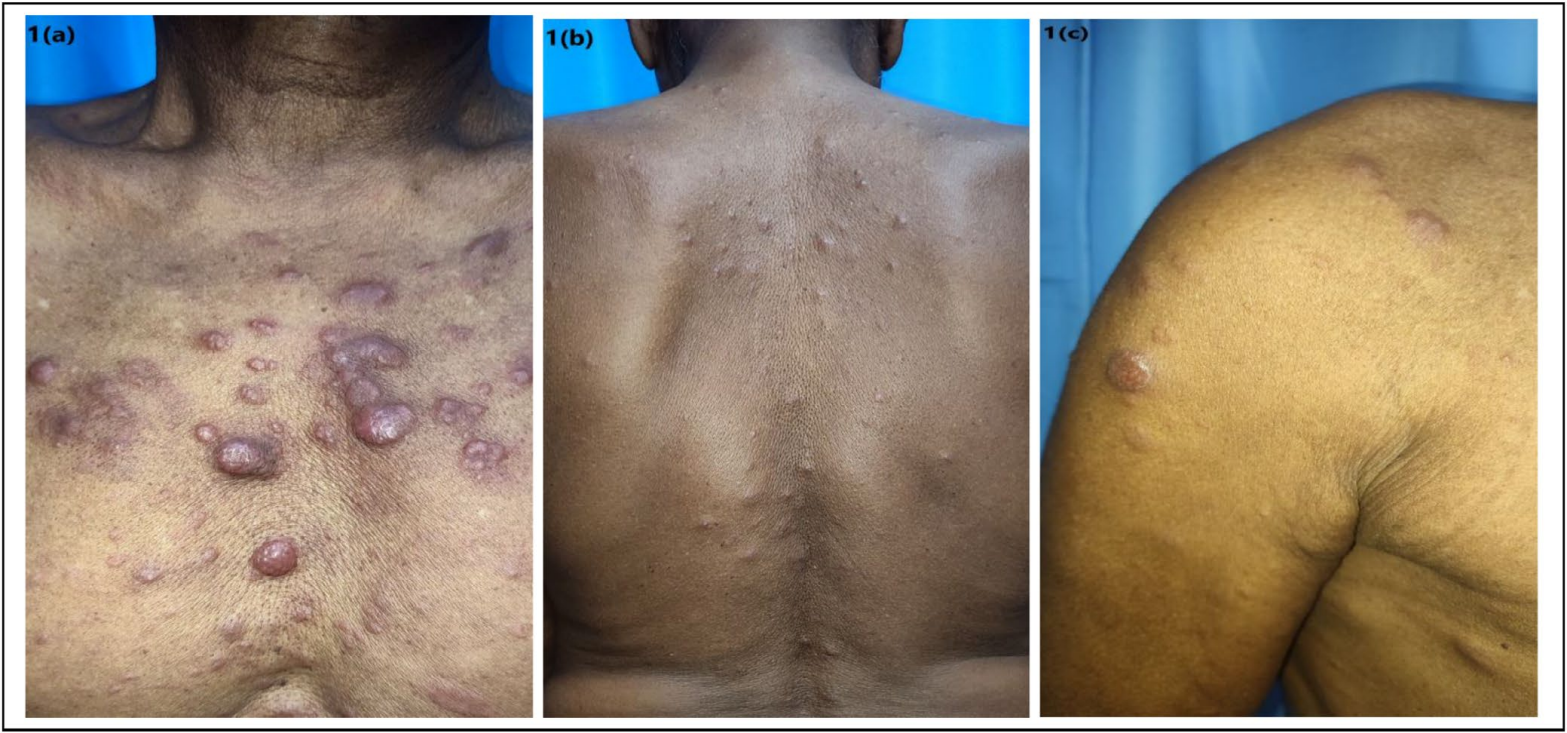
Cutaneous metastasis: (a) Multiple, firm, erythematous papules and plaques over chest, (b) Over posterior trunk, and (c) Over right shoulder and arm.

A 72‐year‐old male patient, diagnosed with NHL of the right testis 8 years back, treated with 6 cycles of chemotherapy (2 cycles of the R‐CHOP regimen and 4 cycles CHOP regimen) along with right‐sided orchidectomy and achieved remission, presented at our center with asymptomatic, multiple, discrete, erythematous papules noticed for the last 3 days. The lesion first appeared on the chest (Figure 1a), which rapidly progressed to involve the posterior trunk and upper extremities (Figure 1b,c). There was a history of anorexia, weight loss, and fatigue for 3 months along with 2 episodes of fever and night sweats 15 days back. On physical examination, generalized lymphadenopathy was observed.

## 3 Differential Diagnosis

Based on history and clinical examinations, cutaneous metastasis, plaque stage of lupus vulgaris, and sarcoidosis were kept as differential diagnoses. Later, the diagnosis was confirmed by skin biopsy.

## 4 Investigations

Right testicular biopsy previously revealed NHL, and IHC markers were consistent with DLBCL. The neoplastic cells were positive for CD20, CD10, BCL2, and MUM‐1, with the c‐MYC marker expressed in more than 40% of cells, suggesting the aggressive nature of the tumor and treatment resistance.

**FIGURE 2:**
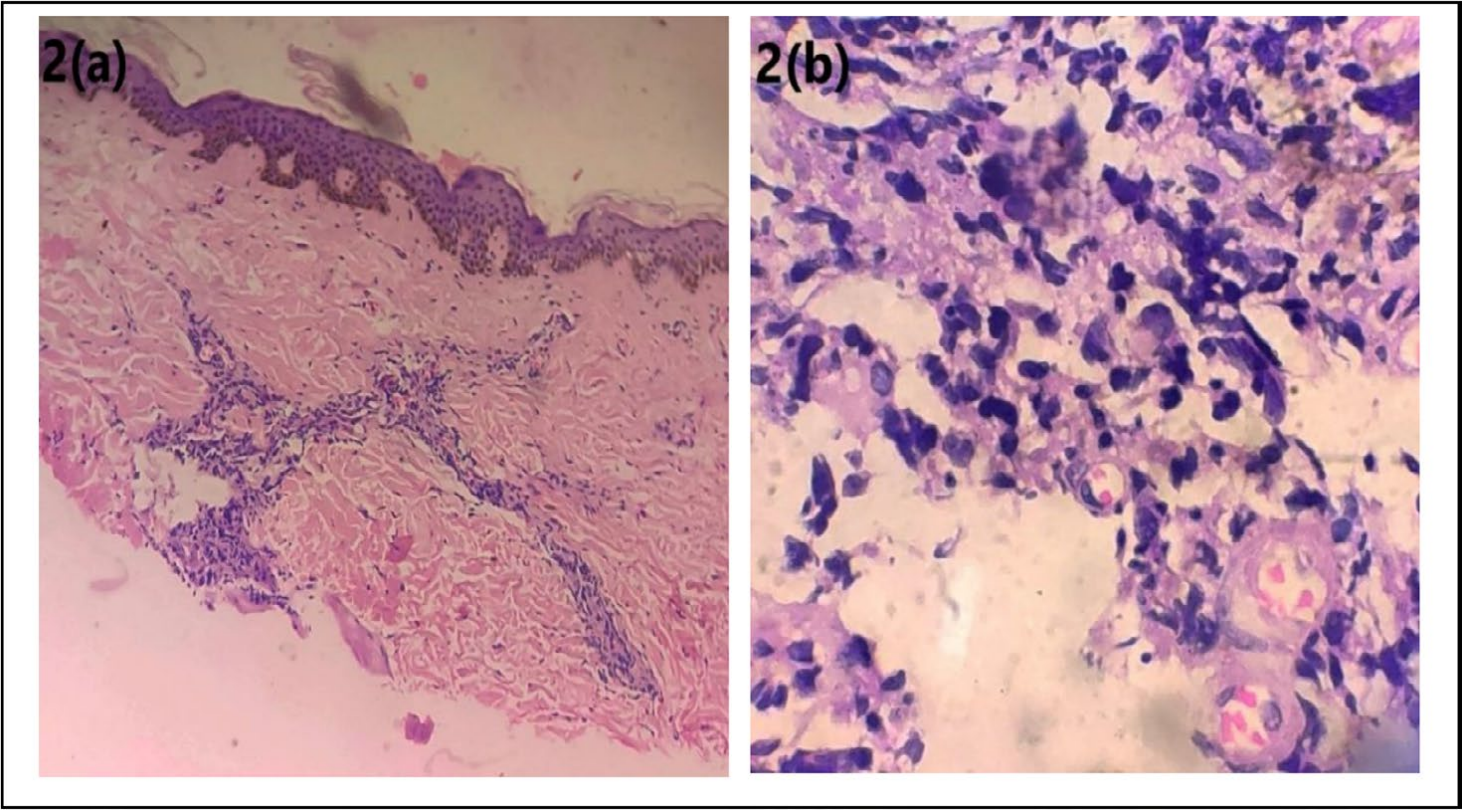
Cutaneous metastasis: (a) Hematoxylin and eosin (H/E) stain 10×: Numerous atypical lymphoid cells in lower dermis, (b) Hematoxylin and eosin (H/E) stain 100×: Numerous atypical cells in dermis with nucleomegaly, irregular nuclear contour and hyperchromasia.

As the patient presented to our center, HPE of a skin biopsy taken from the posterior trunk revealed multiple atypical lymphoid cells in the dermis (Figure 2a,b), and IHC markers were consistent with high‐grade B‐cell non‐Hodgkin's lymphoma, positive for CD20, BCL6, MUM1, and PAX5.

**FIGURE 3:**
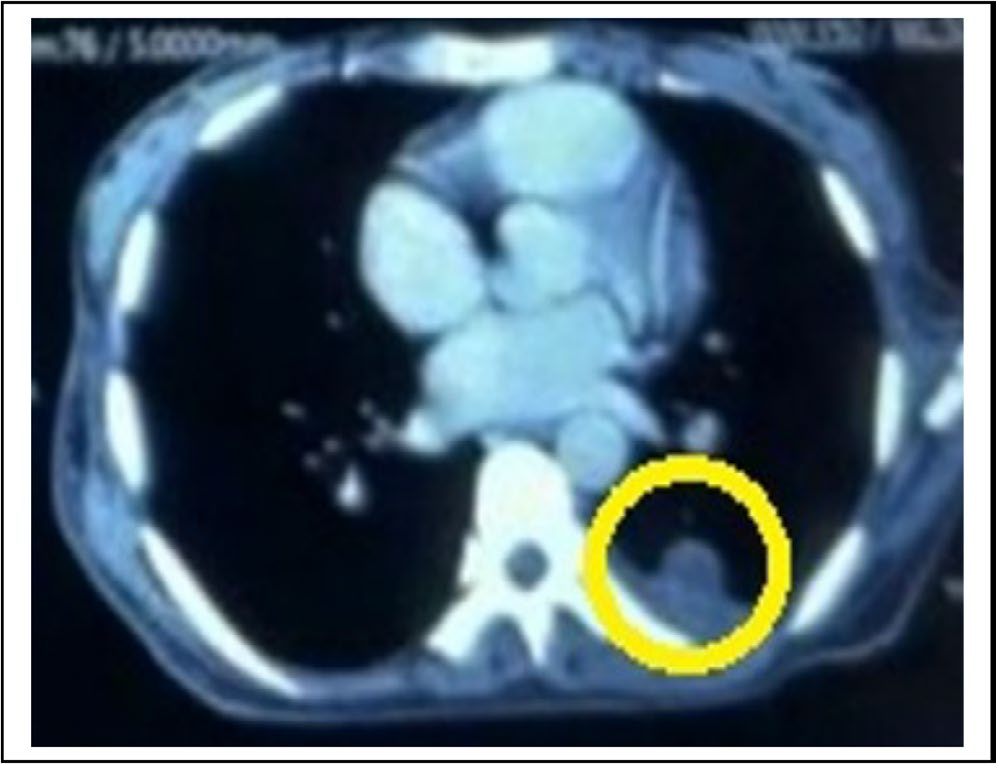
CECT of chest with lung metastasis (yellow circle).

Routine baseline investigations were within normal limits. However, serum lactate dehydrogenase (LDH) was significantly raised (> 3 times the upper normal range). Contrast‐enhanced computed tomography (CECT) of the chest showed features of lung metastasis (Figure 3), mediastinal, and bilateral axillary lymphadenopathy. CECT of the abdomen, pelvis, and neck revealed left inguinal and cervical lymphadenopathy, respectively.

## 5 Outcome and Follow‐Up

Based on the clinical, histopathological, and radiographical findings, the patient was diagnosed to have cutaneous and visceral metastasis secondary to relapse of testicular lymphoma. The patient was referred to a cancer center for further management and is under regular follow‐up. He received 2 cycles of the CHOP regimen. The clinical status of the patient is deteriorating.

## 6 Discussion

Diffuse large B‐cell lymphoma typically expresses pan‐B markers CD19, CD20, CD22, CD79a, CD45RA, PAX5 markers, and other markers CD10, BCL6, BCL2, MYC, and MUM‐1 [4]. In our case, similar IHC markers from the right testis performed 8 years back and the current skin biopsy suggested lymphoma of B cell origin and cutaneous as well as visceral metastasis of testicular B cell lymphoma. Relapse in testicular B cell lymphoma usually involves the contralateral testis and CNS, occurring within 1–2 years from diagnosis of primary lymphoma [4]. However, in our case, relapse occurred late, and the contralateral testis and CNS were not involved.

Mikami T et al. in 2020 reported a case of cutaneous metastasis from testicular diffuse large B‐cell lymphoma and Bowen disease with 18F‐FDG PET‐CT findings. The case report revealed skin metastasis occurring after 1 month of orchidectomy [1]. Varsha Dalal et al. in 2015 reported a case of primary testicular lymphoma with a solitary cutaneous nodule as the initial presentation. The case report revealed a history of an enlarging testicular mass for 8 months and a metastasized nodule in the anterior abdominal wall for 2 months [2]. Hermi et al. in 2021 reported a case of testicular diffuse large B‐cell lymphoma with cutaneous metastasis. The case report revealed a history of a right testicular mass for 3 months, which metastasized to the right inguinal region in 1 month [3]. Likewise, Langar et al. in 2010 also reported a case of left testicular diffuse large B‐cell lymphoma who was in remission following left‐sided orchidectomy and 8 cycles of CHOP regimen 2 years back. Cutaneous relapse of primary testicular lymphoma occurred, and the patient developed metastatic lesions over the right buttock and left forearm for 2 months [5]. In our case, the patient relapsed late, that is, after 8 years, which makes the case report very unique, as late relapse of testicular lymphoma has not been mentioned in literature to date.

Metastases to skin following the NHL of the testis is a rare condition and may occur typically via lymphatic or sometimes by hematogenous route [3]. The pathogenesis behind metastasis involves the blood‐testis barrier, which is a physiological barrier that limits the effectiveness of chemotherapy drugs reaching tumor cells within the testis [6].

As per the “Ann Arbor lymphoma stage classification,” our patient belongs to stage IV, as CECT findings were suggestive of lung metastasis. After calculating the total score as per the international prognostic index, the patient is stratified as a high‐risk group with a 5‐year overall survival of 26% [7].

Primary cutaneous diffuse large B‐cell lymphoma (PCDLBCL) is an important differential diagnosis of metastatic diffuse large B‐cell lymphoma. Lesions in PCDLBCL originate in skin only with no evidence of systemic involvement, unlike metastatic DLBCL, which arises from a systemic lymphoma and spreads to skin. The classical presentation of PCDLBCL is an infiltrative, solitary or multifocal, erythematous or brown‐colored plaque or nodule, most commonly involving the leg (PCDLBCL‐leg type in 60%–70%). Metastatic DLBCL presents with extracutaneous and systemic symptoms [8, 9].

Acute onset and rapid progression of cutaneous lesions, generalized lymphadenopathy, elevated LDH, and lung metastasis are suggestive of aggressive behavior of the tumor and resistance to treatment [10].

Cutaneous metastasis following esophageal squamous cell carcinoma [11], primary pulmonary lymphoma [12] and Bowen disease [1] have been reported in the literature. There are very few cases reported in the literature regarding cutaneous metastasis following the late relapse of testicular lymphoma. The patient was asymptomatic following the treatment, completely lost the follow‐up for the next 8 years, and landed up at our center with cutaneous as well as visceral metastasis. 18F‐fluorodeoxyglucose positron emission tomography‐computed tomography (18F‐FDG PET‐CT) would further allow detection of cutaneous metastasis and data regarding tumor metabolic activity [1]. However, the imaging could not be performed due to its unavailability at our center.

Diagnosis of cutaneous metastasis in a patient with testicular lymphoma remains a challenge. Any patient with a history of lymphoid malignancy presenting with a cutaneous lesion should be carefully investigated to rule out cutaneous metastasis of the underlying lymphoma or may even be the first sign of visceral cancer.

## Author Contributions

**Mahesh Mathur:** conceptualization, data curation, formal analysis, supervision. **Sumit Paudel:** formal analysis, resources, supervision, validation. **Sambidha Karki:** investigation, supervision, validation, visualization. **Sandhya Regmi:** data curation, methodology, supervision, visualization. **Shilpa Maharjan:** data curation, methodology, resources, software. **Nabita Bhattarai:** conceptualization, methodology, validation, writing – original draft.

## Ethics Statement

Reviewed and approved by Institutional Review Board College of Medical Sciences (IRBCOMS).

## Consent

The authors obtained written consent from the patient for the use of photographs and medical information to be published online and with the understanding that this information may be publicly available and discoverable via search engines. Patient consent forms are not provided to the journal but are retained by the authors.

## Conflicts of Interest

The authors declare no conflicts of interest.

## Data Availability

The data that support the findings of this study are available from the corresponding author upon reasonable request.
